# Lipoprotein(a) and SARS‐CoV‐2 infections: Susceptibility to infections, ischemic heart disease and thromboembolic events

**DOI:** 10.1111/joim.13338

**Published:** 2021-10-29

**Authors:** Silvia Di Maio, Claudia Lamina, Stefan Coassin, Lukas Forer, Reinhard Würzner, Sebastian Schönherr, Florian Kronenberg

**Affiliations:** ^1^ Department of Genetics and Pharmacology Institute of Genetic Epidemiology, Medical University of Innsbruck Innsbruck Austria; ^2^ Department of Hygiene, Microbiology and Public Health Institute of Hygiene & Medical Microbiology Medical University Innsbruck Innsbruck Austria

**Keywords:** ischemic heart disease, lipoprotein(a), SARS‐CoV‐2, thromboembolic events

## Abstract

**Background:**

Comorbidities including ischemic heart disease (IHD) worsen outcomes after SARS‐CoV‐2 infections. High lipoprotein(a) [Lp(a)] concentrations are a strong risk factor for IHD and possibly for thromboembolic events. We therefore evaluated whether SARS‐CoV‐2 infections modify the risk of high Lp(a) concentrations for IHD or thromboembolic events during the first 8.5 months follow‐up of the pandemic.

**Method:**

Cohort study using data from the UK Biobank during the SARS‐CoV‐2 pandemic. Baseline Lp(a) was compared between SARS‐CoV‐2 positive patients and the population controls.

**Results:**

SARS‐CoV‐2 positive patients had Lp(a) concentrations similar to the population controls. The risk for IHD increased with higher Lp(a) concentrations in both, the population controls (*n* = 435,104) and SARS‐CoV‐2 positive patients (*n* = 6937). The causality of the findings was supported by a genetic risk score for Lp(a). A SARS‐CoV‐2 infection modified the association with a steeper increase in risk for infected patients (interaction *p*‐value = 0.03). Although SARS‐CoV‐2 positive patients had a five‐times higher frequency of thromboembolic events compared to the population controls (1.53% vs. 0.31%), the risk was not influenced by Lp(a).

**Conclusions:**

SARS‐CoV‐2 infections enforce the association between high Lp(a) and IHD but the risk for thromboembolic events is not influenced by Lp(a).

## Graphical abstract

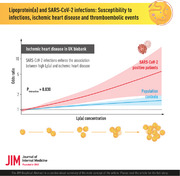

## Introduction

As of April 1, 2021, the confirmed global cases of SARS‐CoV‐2 infections were 128,223,872. The disease is caused by the SARS‐CoV‐2 virus with large variability in susceptibility and severity upon viral exposure. A cross‐sectional survey of 20,133 patients hospitalized with SARS‐CoV‐2 described that patients with diabetes, ischemic heart diseases, hypertension or chronic respiratory diseases are at higher risk of death after a SARS‐CoV‐2 infection [1]. Although the major adverse outcomes are acute respiratory distress syndrome and end‐organ failure, several studies reported a widely varying high frequency of thromboembolic complications which might contribute to end‐organ failure (discussed in Connors et al. [2]). However, population‐based data are limited [3].

The search for suspects which might increase the risk for ischemic heart disease (IHD) and thromboembolic events has set the stage for lipoprotein(a) [Lp(a)], which shows atherogenic and thrombogenic properties [4, 5, 6, 7]. Apolipoprotein(a) is the key protein of this lipoprotein and shows a high homology with plasminogen. Experimental studies provided evidence that high Lp(a) concentrations might promote thrombosis by stimulating platelet activation and coagulation as well as inhibiting fibrinolysis [5]. The inflammatory properties of Lp(a) and its influence on the vascular wall [8] might additionally enhance the thromboinflammatory response to SARS‐CoV‐2 infection [9]. For this and other reasons, Lp(a) was soon after the start of the pandemic discussed as a suspect which could promote IHD and especially thromboembolic events [10, 11]. However, no data have been provided till now.

The present study uses data from the general population of the UK Biobank (UKB) to explore the frequency of IHD and thromboembolic events in SARS‐CoV‐2 positive patients compared to population controls and whether Lp(a) is a risk modulator for these events in case of a SARS‐CoV‐2 infection.

## Materials and methods

### Study cohort

UKB is a large‐scale prospective study with more than 500,000 participants aged 40–69 years at recruitment (2006–2010). UKB received ethical approval from the North West Multi‐Centre Research Ethics Committee (REC reference: 11/NW/0382). All participants gave written informed consent before enrolment in the study, which was conducted in accordance with the principles of the Declaration of Helsinki.

**Fig. 1:**
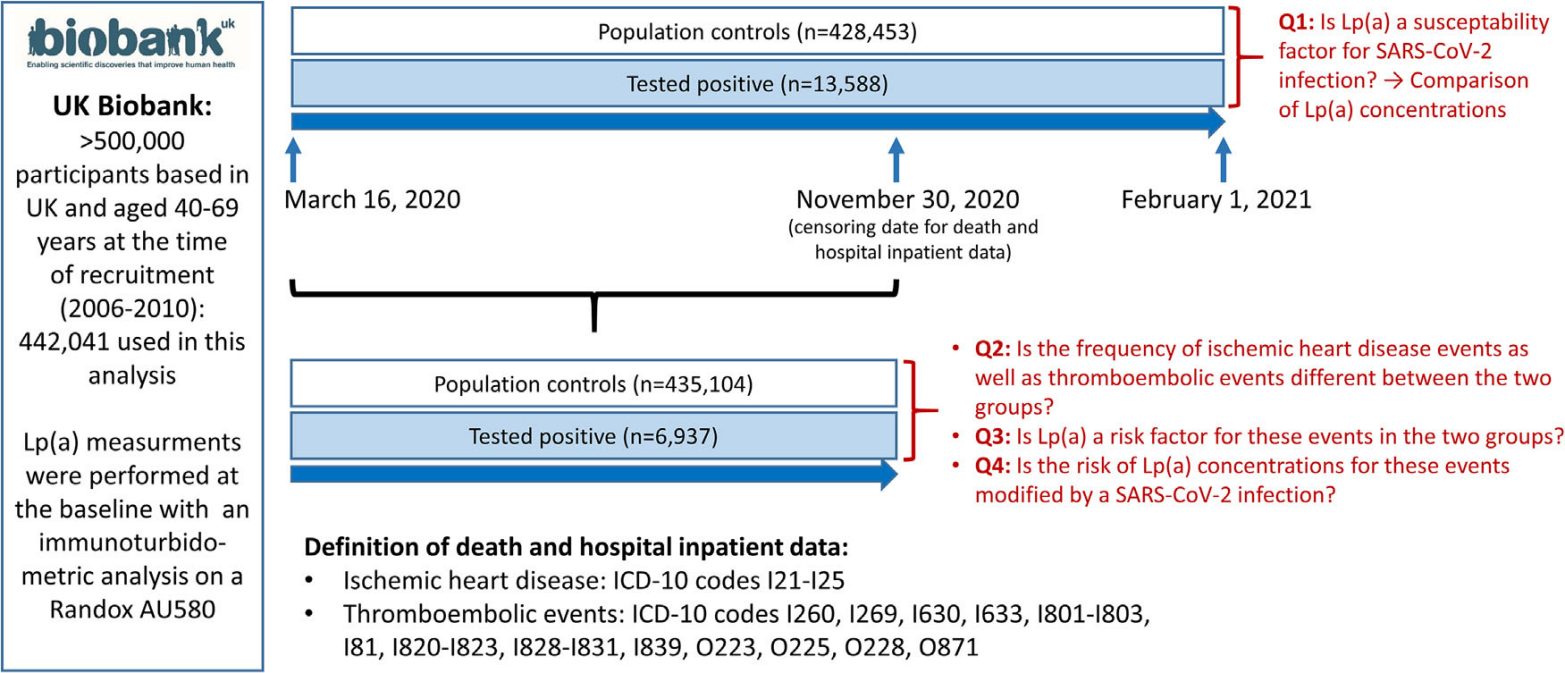
*Description of the study population, study design and research questions (Q1–Q4): The study includes only study participants with Lp(a) measurements available at baseline. For Q1 test results, all participants were included who became available from 16 March 2020 until 1 February 2021. For Q2–Q4, outcome data were available from 16 March 2020 to 30 November 2020. Therefore, the number of tested individuals is lower for this part of the study, and the number of the background population is therefore higher as for Q1*.

Figure 1 and the Supporting Information Material describe the design and research questions of the present investigation. From 16 March 2020 on Public Health England regularly provides UKB with SARS‐CoV‐2 test results [12] (mainly PCR test from combined nose/throat swabs), covering 55,199 individuals tested until 1 February 2021. From participants with Lp(a) measurements available, 13,588 cases were tested positive for SARS‐CoV‐2 at least once in this time frame. As described recently [13], this group was compared to 428,453 population controls which included any person who was not a case (i.e., people who were tested negative, were never tested or had an unknown testing status). A detailed justification of this grouping, a baseline description of the cohort, the phenotyping and statistical analysis are provided in the Supporting Information.

### Outcome data

SARS‐CoV‐2 test results were linked to UKB data with the most complete update on death and hospital inpatient data until 30 November 2020 (censoring date). The outcome data are available for 6937 patients tested positive before the censoring date, of whom 208 required treatment at the intensive‐care unit (ICU), and for 435,104 population controls (either nontested or tested negative before the censoring date) (Table S1). We analysed IHD and thromboembolic events that occurred between 16 March and 30 November 2020 (Fig. 1). IHD events were defined by ICD‐10 codes I21–I25 and thromboembolic events by ICD‐10 codes I260, I269, I630, I633, I801–I803, I81, I820–I823, I828–I831, I839, O223, O225, O228 and O871. To consider only events which are possibly linked to Sars‐CoV‐2 infection in positive tested persons, only events were considered, which occurred after or not more than 10 days before the first positive test.

### Lp(a) concentrations and genetic risk score for Lp(a)

As described by Patel et al. [14], serum Lp(a) concentrations were measured using an immunoturbidimetric assay (Randox Laboratories) (see also Supporting Information).

Two genetic risk scores for Lp(a) were derived: the first one is based on 11,446 variants within 2 Mb of the *LPA* gene [15]. Since this score was developed and validated using the UKB data, another score, not involving UKB, was also used. The second score comprisesing 48 genome‐wide significant SNPs, derived from a genome‐wide meta‐analysis on Lp(a), adjusted for age and sex [16]. They represent a comparable genetic region, spanning 1.76 Mb surrounding the *LPA* locus. In UKB, 850,000 variants were genotyped using the UKB Axiom Array. More than 90 million variants were imputed using the Haplotype Reference Consortium and UK10K + 1000 Genomes reference panels. SNPs for the genetic risk scores were retrieved from these data. Each genotype dosage was multiplied by the corresponding weights, and all values were summed up to the individual score using PGS‐Calc (https://github.com/lukfor/pgs‐calc).

### Statistical analysis

The difference in Lp(a) levels between groups was tested using Wilcoxon test. Frequencies in IHD and thromboembolic events between groups were tested using chi‐square test. Logistic regression analyses were performed to estimate the risk of Lp(a) increase on positive status for SARS‐CoV‐2 infection, IHD and thromboembolic events. The main analysis was adjusted for age, sex, ethnicity, smoking, body mass index, diabetes mellitus, hypertension and LDL cholesterol. A further model was conducted additionally adjusting for prevalent IHD (including prevalent case status until September 2019). Lp(a) was used both as a continuous variable (estimates given for each 25 nmol/L increase) and in categories for IHD and thromboembolic events: <20th percentile (reference category), 6.1–75, 75–120, 120–220 and >220 nmol/L ( = 95th percentile). Nonlinear splines were plotted using the function ‘gam’ in the package ‘mgcv’ (R version 4.0.3.) to evaluate a possible nonlinear relationship of Lp(a) on outcomes. We tested for interaction between continuous Lp(a) levels and the grouping variable ‘population controls versus SARS‐CoV‐2 positive tested’.

## Results

### Lp(a) concentrations in SARS‐CoV‐2 positive patients

First, we investigated whether Lp(a) is a susceptibility factor for SARS‐CoV‐2 infection. We observed no difference in the Lp(a) distribution between SARS‐CoV‐2 positive patients and the population controls (Fig. S1, median Lp(a) concentrations 19.55 and 19.60 nmol/L, respectively, *p* = 0.38). The results did not change when we adjusted for age, sex, ethnic background, BMI, diabetes, hypertension, LDL cholesterol and smoking status using a logistic regression model (Table S2).

### Lp(a) and IHD

**Fig. 2:**
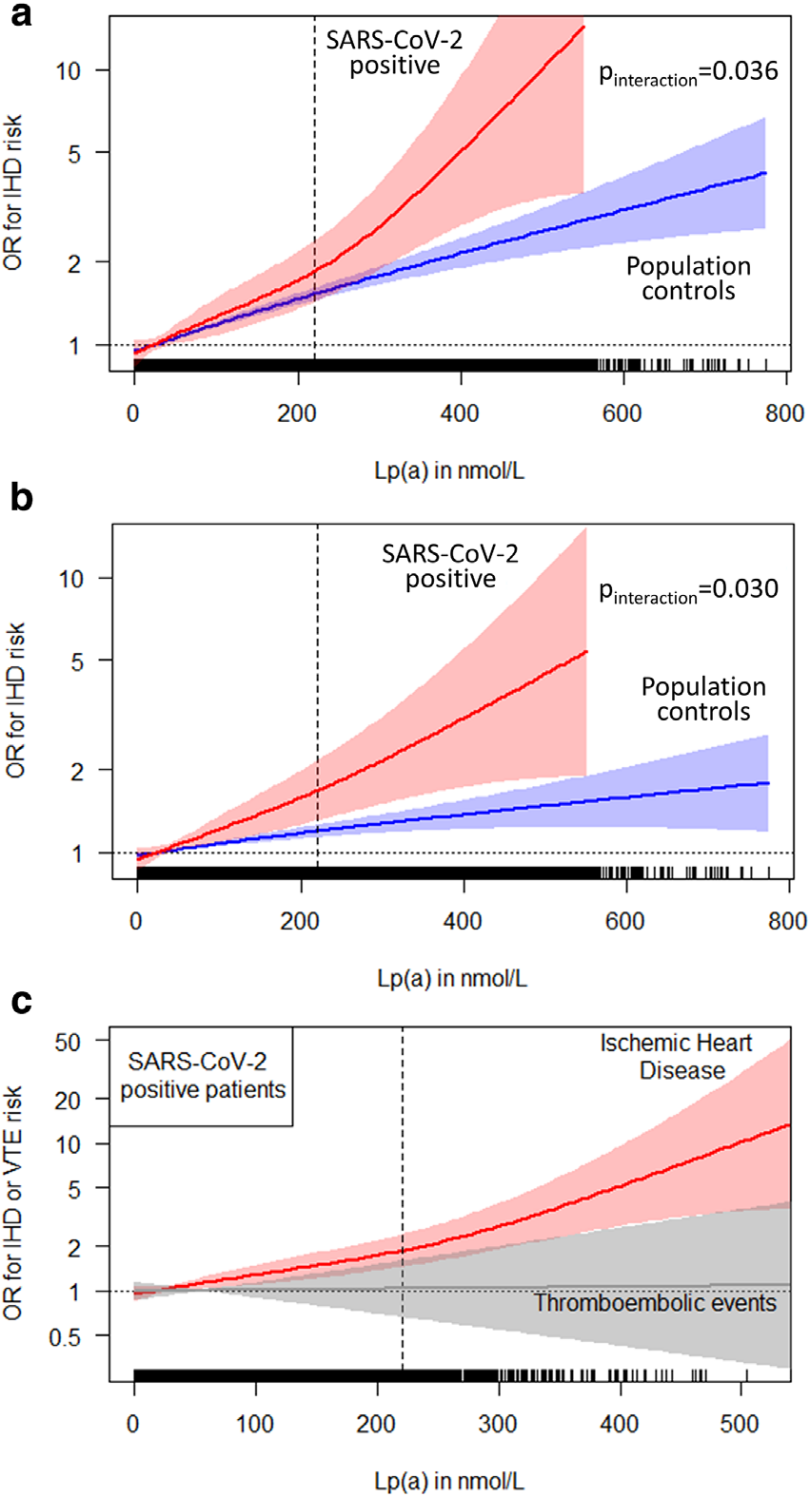
*(a) Nonlinear splines describing the association between Lp(a) concentrations and ischemic heart disease (IHD) events in SARS‐CoV‐2 positive patients and in the population controls. Data are adjusted for age, sex, ethnicity, smoking, body mass index, diabetes mellitus, hypertension and Lp(a)‐corrected LDL cholesterol*.p*‐value for interaction = 0.036. The dotted line for OR = 1 crosses the spline at the Lp(a) median value of 19.6 nmol/L in all analysed individuals. The dashed vertical line corresponds to an Lp(a) level of 220 nmol/L (= 95th percentile). (b) is similar to panel (a) but additionally adjusted for prevalent IHD status from September 2019*. p*‐Value for interaction = 0.030. (c) Splines for IHD and venous thromboembolism outcomes in 6937 SARS‐CoV‐2 positive tested study participants. The dotted line for OR = 1 crosses the spline at the Lp(a) median value of 19.2 nmol/L. The dashed vertical line corresponds to an Lp(a) level of 220 nmol/L (= 95th percentile). In each plot, tick marks at the bottom line indicate one observation*.

The IHD frequency in SARS‐CoV‐2 positive patients, either hospitalized or not, was markedly higher than in the population controls (4.67% vs. 1.48%, *p* = 1.84e–106). In the population controls, we observed the expected pattern of increasing IHD risk with increasing Lp(a) concentrations with an OR of 1.04 for each 25 nmol/L increase of Lp(a) (95% CI 1.05–1.06, *p* < 2e–16). This risk increase was even steeper in SARS‐CoV‐2 positive patients (OR = 1.07, 95% CI 1.03–1.10, *p* = 0.00026) with a significant interaction between SARS‐CoV‐2 positive status and Lp(a) levels (*p* = 0.036). Indeed, the OR for IHD of the top 5% compared to the bottom 20% of the Lp(a) concentrations was 48% higher in SARS‐CoV‐2 positive patients compared to the population controls (OR 2.22 versus 1.50; Table 1). Figure 2 shows that there was no indication for nonlinearity in the association of Lp(a) on IHD risk. It also underscores the steeper risk increase in the positive tested group (Fig. 2a, *p*‐value for interaction = 0.0036). The difference remained unchanged when we adjusted for the status of prevalent IHD before the pandemic started (Fig. 2b, *p*‐value for interaction = 0.030).

**Table 1:** *Logistic regression for ischemic heart disease events as well as thromboembolic events in population controls (reflecting the general population) (*n *= 435,104) and SARS‐CoV‐2 positive tested patients (*n *= 6937). Observation period: 16 March–30 November 2020. Data are odds ratios, 95% confidence intervals*, p*‐values and* N *reflect the number of patients with events/number of individuals at risk*

	Lp(a) ≤6.1 nmol/L = 20th percentile	Lp(a) >6.1– <75 nmol/L	Lp(a) ≥75– <120 nmol/L	Lp(a) ≥120– 220 nmol/L	Lp(a) >220 nmol/L = 95th percentile	Lp(a) >95th versus <20th percentile
**Ischemic heart disease**						Comparison within the groups*
Population controls	1.00 Reference *N* = 1167/87,289	1.06 (0.99–1.14) *p* = 0.09 *N* = 3144/239,333	1.27 (1.14–1.41) *p* = 1.27e–05 *N* = 548/33,628	1.30 (1.19–1.42) *p* = 2.01e–08 *N* = 953/53,099	1.50 (1.34–1.69) *p* = 2.20e–12 *N* = 489/21,755	1.50 (1.34–1.69) *p* = 2.20e–12
SARS‐CoV‐2 positive tested patients	3.25 (2.47–4.29) *p* < 2e–16 *N* = 62/1428	3.35 (2.78–4.03) *p* < 2e–16 *N* = 150/3634	5.56 (3.81–8.10) *p* < 2e–16 *N* = 33/517	4.02 (2.88–5.61) *p* = 2.74e–16 *N* = 42/807	7.24 (4.96–10.61) *p* < 2e–16 *N* = 37/343	2.22 (1.40–3.54)* *p* = 0.00067
**Thromboembolic events**						
Population controls	1.00 Reference *N* = 247/87,289	1.08 (0.93–1.25) *p* = 0.29 *N* = 723/239,333	1.08 (0.85–1.38) *p* = 0.53 *N* = 94/33,628	1.08 (0.88–1.32) *p* = 0.48 *N* = 162/53,099	1.29 (1.00–1.68) *p* = 0.051 *N* = 78/21,755	1.29 (1.00–1.68) *p* = 0.051
SARS‐CoV‐2 positive tested patients	6.90 (4.58–10.41) *p* < 2e–16 *N* = 29/1476	5.39 (3.96–7.34) *p* < 2e–16 *N* = 54/3741	4.75 (2.09–10.77) *p* = 0.0002 *N* = 8/534	5.17 (2.81–9.53) *p* = 1.38e–07 *N* = 11/826	4.98 (2.03–12.12) *p* = 0.0004 *N* = 5/360	0.72 (0.27–1.90)* *p* = 0.51

*Note*: Data are adjusted for age, sex, ethnicity, smoking, body mass index, diabetes mellitus, hypertension and Lp(a)‐corrected LDL cholesterol.

* This analysis uses as reference SARS‐CoV‐2 positive patients with Lp(a) below the 20th percentile and no longer those from the population controls.

When we repeated the analysis stratifying patients by the median of 57 years of age at the time of recruitment, we observed an association of Lp(a) with IHD in both groups, which was stronger in the younger age group (Table S3).

### Lp(a) and thromboembolic events

We analysed thromboembolic events during the observation period. These were five‐times more frequent in positive patients when compared to the population controls (1.54% vs. 0.31%, *p* = 4.68e–74). Within the cohort of 6937 positively tested patients before the censoring date, 3.0% required ICU treatment. The incidence of thromboembolic events was markedly higher in ICU‐treated patients compared to SARS‐CoV‐2 positive patients without ICU treatment (10.60% vs. 1.30%; *p* = 6.95e–27). However, we did not observe any association between Lp(a) and thromboembolic events as can be seen by comparing the ORs in the various Lp(a) strata (Table 1) and in Fig. 2c. Within SARS‐CoV‐2 positive patients, there was absolutely no association when we performed a logistic regression model adjusted for age, sex, BMI, diabetes, ethnicity, smoking status and hypertension (OR = 1.00, *p* = 0.90) and when we performed a spline analysis over the entire range of Lp(a) concentrations (Fig. 2c).

### Application of an Lp(a) genetic risk score

Both Lp(a) genetic risk scores derived from different populations (15, 16) did not differ between the SARS‐CoV‐2 positive patients and the population controls (both *p* > 0.30) and were also not associated with thromboembolic events (*p* > 0.41). However, the Lp(a) genetic risk scores increased the risk for IHD in the entire population (*p* < 2e–16).

## Discussion

Using data from the UKB, we could show that the frequency of thromboembolic events is increased in SARS‐CoV‐2 positive patients and especially in those requiring intensive‐care treatment. High Lp(a) concentrations do not increase the susceptibility to SARS‐CoV‐2 infections nor to thromboembolic events. However, Lp(a) is a risk factor for IHD events and – most importantly – the association of high Lp(a) with IHD risk is even stronger under the circumstances of a SARS‐CoV‐2 infection.

Soon after the COVID‐19 pandemic started, it became obvious that thromboembolic events are a frequent complication that contributes to the end‐organ damages in these patients. However, the reported frequencies varied widely from a few percentage in hospitalized patients to more than 30% in intensive care patients [17, 18]. Piazza et al. reported a 2.6% frequency of major arterial and venous thromboembolism and a 2.2% frequency of symptomatic venous thromboembolism in a cohort of 229 hospitalized nonintensive care cohort. This frequency was 35.3% and 27.0%, respectively, in 170 intensive care patients [17]. Another study involving hospitalized SARS‐CoV‐2 patients reported a venous thromboembolism incidence of 7.2% and 3.1% in patients with and without need for mechanical ventilation, respectively [18]. A recent population‐based registry from Denmark reported an overall 30‐day risk of venous thromboembolism of 0.4% and 0.3% for SARS‐CoV‐2 positive and SARS‐CoV‐2 negative patients with a risk of 1.8% and 1.5% when patients were hospitalized. In the same publication, a medical record review from 582 hospitalized SARS‐CoV‐2 patients revealed 4% and 7% frequencies of patients at the ward and intensive care unit, respectively [3]. In the present UKB data, the observed events rates were roughly fourfold and 30‐fold higher in SARS‐CoV‐2 positive patients without and with treatment at the ICU, respectively, when compared to the population controls (1.30% and 10.6% vs. 0.31%), but the frequencies were still lower than in some earlier reports. One reason for this might be that UKB cohort is closer to a population‐based sample which might avoid an overestimation of the frequency. A further explanation could be a possible change in the anticoagulation strategy in the treatment of severe SARS‐CoV‐2 infections compared to the first wave of SARS‐CoV‐2 infections, as it became clear that thromboembolic events represented a major adverse outcome.

The present data do not provide any evidence that high Lp(a) concentrations trigger an increased risk for thromboembolic events in case of a SARS‐CoV‐2 infection as suggested recently [10, 11]. This was at the first glance surprising. However, most of the current evidence for a thrombogenic nature of Lp(a) derived from experimental studies. Epidemiologic and genetic studies did not provide support for a thrombogenic role and if there is any, it can only be found at very high Lp(a) values above the 95th percentile [19]. Similarly, we observed in the population controls a tendency of an increased risk for thromboembolic concentrations (HR = 1.29, *p* = 0.051). Interestingly, even under these circumstances of an increased frequency of thromboembolic events in SARS‐CoV‐2 positive patients, Lp(a) did not modify the risk.

The most important finding of our analysis is the observation that high Lp(a) concentrations further increase the risk for IHD in SARS‐CoV‐2 positive patients compared to the population controls, where the risk was increased already when Lp(a) concentrations were above 75 nmol/L (OR = 1.27, *p* = 1.27e–05). Since the outcome of patients with pre‐existing IHD is worse in case of a SARS‐CoV‐2 infection [1], a high Lp(a) concentration with an accompanying inflammation of the arterial wall together with an exacerbated immune reaction might further enhance IHD risk [20]. Patients with high Lp(a) concentrations might therefore be considered as high‐risk group in case of a SARS‐CoV‐2 infection especially when they had already suffered an IHD event in the past.

Our study has strengths and limitations. Strengths are an almost population‐based cohort with Lp(a) measurements long before the SARS‐CoV‐2 pandemic. This excludes reverse causation meaning that the disease itself is changing Lp(a) concentrations and thereby influencing the finding. Furthermore, it is one of the very few studies which comes close to population‐based data and might provide a more realistic estimate on the thromboembolic event estimates in case of a SARS‐CoV‐2 infection. The study is limited by the circumstances that we do not have detailed data on the treatment of the patients during hospitalization and how this might have influenced our finding. We furthermore cannot exclude that short‐term changes of Lp(a) during the acute phase could have influenced the findings.

## Conclusions

High Lp(a) concentrations do not seem to increase the risk for thromboembolic events in SARS‐CoV‐2 positive patients. However, high Lp(a) concentrations are an even stronger risk factor for IHD under the circumstance of a SARS‐CoV‐2 infection compared to population controls. Therefore, patients with high Lp(a) concentrations might be considered as high‐risk group in case of a SARS‐CoV‐2 infection.

## Conflict of interest

The authors declare no competing interests.

## Supporting information

Supporting information
