# Supplementary material for: Lipoprotein(a) and SARS‐CoV‐2 infections: Susceptibility to infections, ischemic heart disease and thromboembolic events
Source: J Intern Med. 2021 Oct 29;291(1):101–7. doi: 10.1111/joim.13338 (PMC8242884; doi:10.1111/joim.13338)

Supplementary Material

Lipoprotein(a) and SARS-CoV-2 infections: susceptibility to infections, ischemic heart disease and thromboembolic events

Silvia Di Maio [1], MSc, Claudia Lamina [1], PhD, Stefan Coassin [1], PhD, Lukas Forer [1], PhD, Reinhard Würzner [2], MD, PhD, Sebastian Schönherr [1], PhD, Florian Kronenberg [1], MD

[1] Institute of Genetic Epidemiology, Department of Genetics and Pharmacology, Medical University of Innsbruck, Innsbruck, Austria

[2] Institute of Hygiene & Medical Microbiology, Department of Hygiene, Microbiology and Public Health, Medical University Innsbruck, Innsbruck, Austria

Corresponding author:

Florian Kronenberg, MD
Institute of Genetic Epidemiology
Department of Genetics and Pharmacology
Medical University of Innsbruck
Schöpfstrasse 41, A-6020 Innsbruck, Austria.
Phone: (+43) 512-9003-70560
Fax: (+43) 512 9003-73560 or -73561,
Email: Florian.Kronenberg@i-med.ac.at

Supplementary Methods

Definition of the control group and the SARS-CoV-2 positive patient group

UKB is a large-scale prospective study with more than 500,000 participants aged 40-69 years at recruitment (2006-2010). From March 16, 2020 on Public Health England regularly provides UKB with SARS-CoV-2 test results [11] (mainly PCR test from combined nose/throat swabs), covering 55,199 individuals tested until February 1, 2021. From participants with Lp(a) measurements available, 13,588 cases were tested positive for SARS-CoV-2 at least once up to this date. As described recently [1, 2] the positive-tested case group was compared to the population controls, which included any person who was not a case (i.e., people who were tested negative, were never tested, or had an unknown testing status). The reason for building this control population is as follows: during the first months of the pandemic, COVID-19 testing was not so widely used as during the second and third wave of the pandemic and was therefore mainly limited to hospital inpatients. A major fraction who got tested was the part of the population who had to be hospitalized or had to search help from the health care system. Therefore, we expected that a major part of the tested persons are those with diseases which have nothing to do with a SARS-CoV-2 infection. These individuals are therefore an important part of the "control" population. This would result in a biased enrichment of patients with e.g. cardiovascular events in the negative-tested patients. This can be best seen by the fact, that the ischemic heart disease events were 0.7% in the non-tested group but 11.2% in the negative-tested group. For the thrombotic events, the rate was 0.1% in the non-tested group but 2.4% in the negative-tested group. The same approach for building the control group has been used in recent publications [1, 2]. Building this type of a control group could have resulted in a misclassification in case a fraction of the non-tested part of the population might have undergone a SARS-CoV-2 infection without symptoms with the following two possible scenarios: if these asymptomatic patients would have developed more often cardiovascular events compared to those who remained indeed SARS-CoV-2 negative, this would have diluted our results meaning that the differences compared to the positive patients would have been biased towards the zero. If the asymptomatic negative-tested subjects would have the same risk for ischemic heart disease events, then the observed results would not change. In any way, our observed estimates might therefore be more on the conservative side.

Definition of phenotypes

Hypertension was present when the systolic blood pressure was 140 mm Hg or higher or when the diastolic blood pressure was 90 mm Hg or higher or when the study participant was taking antihypertensive drugs with the ATC codes C02 (antihypertensives), C03 (diuretics), C07 (betablockers), C08 (calcium channel blockers) or C09 (ACE inhibitors, angiotensin receptor blockers).

A study participant has been considered to have diabetes mellitus if HbA1c in percent was ≥6.5% or when antidiabetic medications with the ATC code A10 were taken. In UKBB HbA1c is given in mmol/mol. To convert to percent: (HbA1c[mmol/mol] / 10.929) + 2.15.

Lp(a) measurement

As described in the publication by Patel et al. [3], serum Lp(a) concentrations were measured using an immunoturbidimetric assay (Randox Laboratories; Crumlin, County Antrim, United Kingdom using a Beckman Coulter AU5800 Platform). This assay employs the Denka Seiken method, which has been shown to have excellent concordance with reference material from the World Health Organization/International Federation of Clinical Chemistry. 97,789 (17.3%) of these individuals had concentration values outside of the assay's clinically reportable range (3.8–189 nmol/L): 46,836 (58.7%) had values below 3.8 nmol/L, and 32 953 (41.3%) had values above 189 nmol/L. Among individuals in whom Lp(a) was detected above the analytical range, serial dilutions of the sample were carried out, and the sample was reanalysed as described in: https://biobank.ndph.ox.ac.uk/showcase/showcase/docs/serum_biochemistry.pdf.

References

1. Leong A, Cole JB, Brenner LN*, et al.* Cardiometabolic risk factors for COVID-19 susceptibility and severity: A Mendelian randomization analysis. *PLoS Med* 2021; **18:** e1003553.
2. Aung N, Khanji MY, Munroe PB*, et al.* Causal Inference for Genetic Obesity, Cardiometabolic Profile and COVID-19 Susceptibility: A Mendelian Randomization Study. *Front Genet* 2020; **11:** 586308.
3. Patel AP, Wang M, Pirruccello JP*, et al.* Lp(a) (Lipoprotein[a]) Concentrations and Incident Atherosclerotic Cardiovascular Disease: New Insights From a Large National Biobank. *Arterioscler Thromb Vasc Biol* 2021; **41:** 465-474.

Supplementary Table 1: Baseline characteristics of UK Biobank participants. Continuous variable provided as [25th percentile; 50th percentile; 75th percentile]. Categorical variables provided as [absolute number (%)]. The percentage is relative to the total number of participants with the considered variable available. Diabetes status was not available for n=22,506 participants, hypertension for n=363, BMI for n=1630, smoking status for n=458, ethnicity for n=460.

	**SARS-CoV-2 positive (n=6,937)**	**Population controls * (n=435,104)**
Age at recruitment	46; 54; 62	50; 58; 63
Age in year 2020	58; 65; 74	62; 69; 75
Female sex	3,532 (50.9%)	238,615 (54.8%)
White ethnicity	6,351 (91.63%)	409,451 (94.20%)
Diabetes	463 (7.1%)	20,056 (4.9%)
Hypertension	3,613 (52.1%)	235,376 (53.1%)
Prevalent ischemic heart disease at 2019	660 (9.50%)	30,020 (6.9%)
Lp(a) (nmol/L)	7.3; 19.1; 73.1	7.6; 19.6; 74.6
Lp(a)-corrected LDL cholesterol (mg/dL)**	104; 125; 148	107; 129; 152
Body mass index, kg/m²	24.87; 27.73; 30.98	24.11; 26.70; 29.83
Never smokers	3,554 (51.3%)	240,371 (55.3%)

* Population controls included any person who was not a SARS-CoV-2 positive case (i.e., people who were tested negative, were never tested, or had an unknown testing status)

** LDL cholesterol has been corrected for the cholesterol content of Lp(a) (assuming that Lp(a) consists of roughly 30% cholesterol).

Supplementary Table 2: Results of logistic regression analysis investigating the association between Lp(a) concentrations and other factors and susceptibility to a SARS-CoV-2 infection.

	OR	95%CI	P Value
**Lp(a) per 25 nmol/L increment**	**0.997**	**0.991-1.003**	**0.40**
Age	0.970	0.958-0.962	<2e-16
Male Sex	1.05	1.02-1.09	0.005
White ethnicity	0.61]	0.58-0.65	<2e-16
Body mass index, kg/m²	1.038	1.034-1.041	<2e-16
Diabetes	1.22	1.13-1.32	3.3e-07
Hypertension	0.94	0.90-0.98	0.003
Smoking status			
Previous smokers	1.20	1.14-1.27	<2e-16
Current smokers	1.11	1.05-1.18	0.0005
Lp(a)-corrected LDL cholesterol	0.998	0.997-0.999	5.2e-12

Note of caution: The estimates for comorbidities have to be considered with caution since testing strategies have been changing during the pandemic. The tested population does not represent the general UK population. For instance, comorbid conditions increase the probability of being symptomatic and therefore being tested. However, in does not mean that these comorbidities make the person more susceptible for SARS-CoV-2 infections. A recent Mendelian randomization study found only genetically elevated BMI to be associated with susceptibility to SARS-CoV-2 infection [1].

Supplementary Table 3: Logistic regression for ischemic heart disease events in population controls (reflecting the general population) (n=435,104) and SARS-CoV-2 positive tested patients (n=6,937) stratified by the median of age (57 years at the time of recruitment). Observation period: March 16 to November 30, 2020. Data are odds ratios (OR), 95% confidence intervals, P-values, and N reflect the number of patients with events / number of individuals at risk.

	**Lp(a) <6.1 nmol/L = 20th percentile**	**Lp(a) 6.1-75 nmol/L**	**Lp(a) >75 - 120 nmol/L**	**Lp(a) >120 - 220 nmol/L**	**Lp(a) >220 nmol/L = 95th percentile**	**Lp(a) >95th versus <20th percentile**
**Ischemic heart disease: patients below median of age (57 years)**						**Comparison within the groups ***
Population controls	1.00<br>Reference<br>N = 260 / 42099	1.04 (0.89-1.21)<br>P = 0.62<br>N = 672 / 110101	**1.34 (1.08-1.67)**<br>P = 0.008<br>N = 132 / 16743	**1.34 (1.10-1.63)**<br>P = 0.004<br>N = 187 / 23849	**1.94 (1.51-2.48)**<br>P = 1.56e-07<br>N = 94 / 8548	**1.94 (1.51-2.48)**<br>P = 1.56e-07
SARS-CoV-2 positive tested patients	**1.19 (0.52-2.70)**<br>P = 0.66<br>N = 6 / 891	**2.35 (1.57-3.51)**<br>P < 3.57e-05<br>N = 28 / 2192	**4.13 (1.91-8.92)**<br>P = 0.0003<br>N = 7 / 323	**2.20 (0.90-5.40)**<br>P = 0.08<br>N = 5 / 457	**8.85 (4.22-18.56)**<br>P = 8.19e-09<br>N = 9 / 174	**7.35 (2.47-21.88)**<br>P = 0.000331
**Ischemic heart disease: patients above median of age (57 years)**						
Population controls	1.00<br>Reference<br>N = 907 / 45190	1.07 (0.98-1.15)<br>P = 0.12<br>N = 2472 / 129232	**1.24 (1.10-1.40)**<br>P = 0.0005<br>N 416 / 16885	**1.27 (1.15-1.41)**<br>P = 4.85e-06<br>N = 766 / 29250	**1.37 (1.20-1.55)**<br>P = 2.02e-6<br>N = 395 / 13207	**1.37 (1.20-1.55)**<br>P = 2.02e-6
SARS-CoV-2 positive tested patients	**4.08 (3.03-5.50)**<br>P < 2e-16<br>N = 56 / 585	**3.76 (3.05-4.63)**<br>P < 2e-16<br>N = 122 / 1549	**6.33 (4.09-9.80)**<br>P < 2e-16<br>N = 26 / 204	**4.60 (3.19-6.63)**<br>P = 3.07e-16<br>N = 37 / 369	**6.62 (4.25-10.31)**<br>P < 2e-16<br>N = 28 / 186	1.62 (0.96-2.74)<br>P = 0.071

Supplementary Figure 1: Distribution of the Lp(a) concentrations in participants of the UK Biobank. Panel A shows the distribution in patients tested SARS-CoV-2 positive between March 16, 2020 and February 1, 2021 and Panel B shows the distribution of Lp(a) in population controls. There was no significant difference in Lp(a) concentrations between the two groups (P=0.38). n=1536 individuals had Lp(a) levels >400 nmol/L.

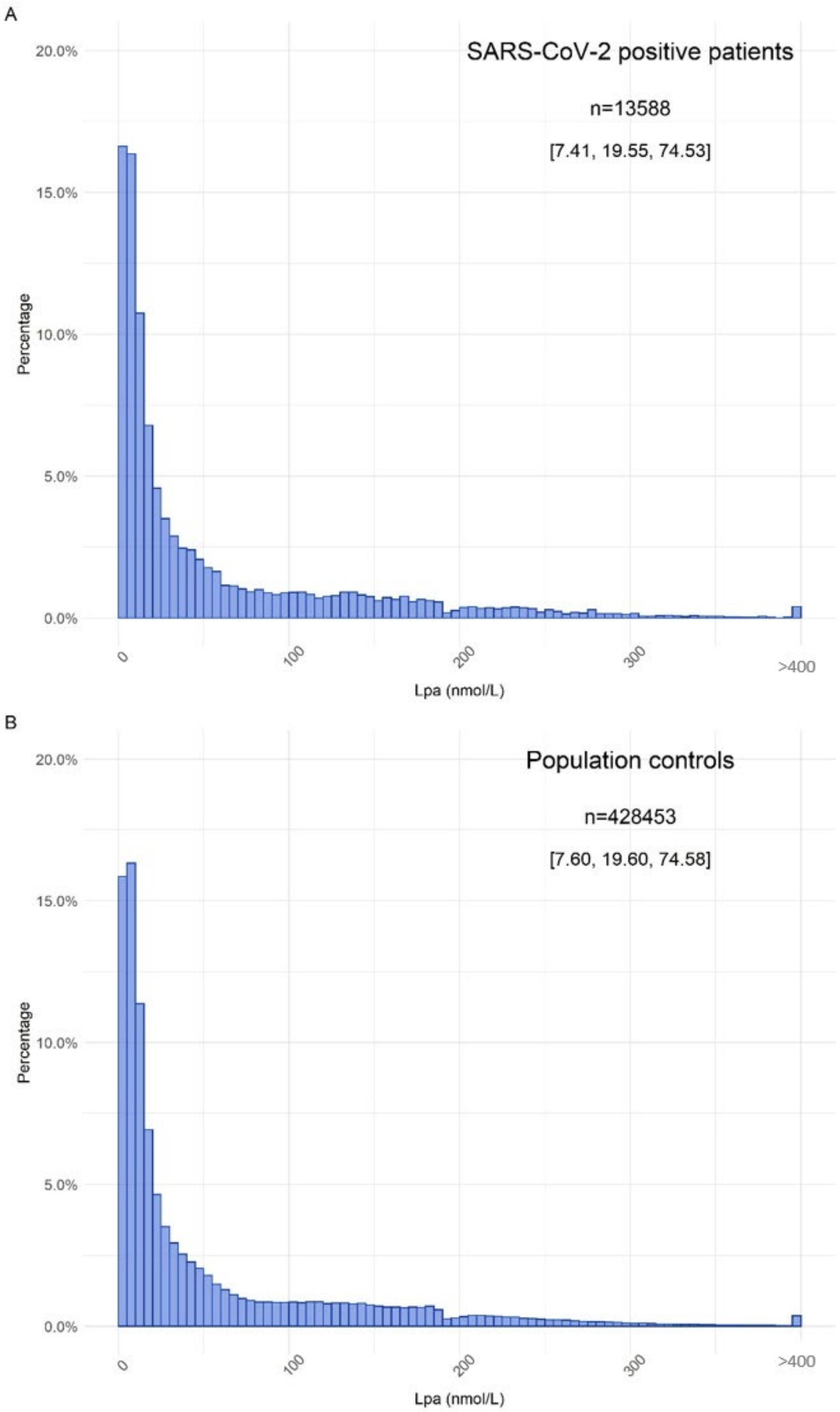

Supplement: Supplementary file 1 — Supporting information [file JOIM-291-101-s001.pdf]
